# From MRI activity to treatment escalation in relapsing multiple sclerosis: what can new T2 lesions tell us?

**DOI:** 10.3389/fneur.2026.1872262

**Published:** 2026-08-25

**Authors:** Stefan Buchka, Ulrich Mansmann, Joachim Havla

**Affiliations:** 1 Institute for Medical Information Processing, Biometry, and Epidemiology, Medical Faculty, Ludwig-Maximilians University Munich, Munich, Germany; 2 Pettenkofer School of Public Health, Medical Faculty, Ludwig-Maximilians University Munich, Munich, Germany; 3 Institute of Clinical Neuroimmunology, LMU University Hospital, LMU Medizin, Ludwig-Maximilians-Universität München, Munich, Germany

**Keywords:** relapsing multiple sclerosis, T2-weighted lesions, treatment decisions, individual risk prediction, prognostic model validation, treatment-effect heterogeneity, decision curve analysis, shared decision-making

## Introduction

Imagine a person with relapsing multiple sclerosis (PwRMS) who is clinically stable 1 year after starting a disease-modifying treatment. A routine brain MRI nevertheless shows three new or enlarged T2-weighted lesions (T2L). The finding should not be ignored: it documents inflammatory activity that was not clinically apparent. However, the new T2L is not necessarily a predictor of the patient's future disability progression.

To move from the MRI finding to a treatment decision, four questions must be separated. First, have new lesions detected breakthrough disease activity? Second, what is the person's future risk of relapse or disability progression if the current management is continued? Third, would an alternative treatment provide greater benefit for this particular person? Fourth, does an MRI-guided escalation strategy improve outcomes when its benefits, harms, and burdens are compared with relevant alternatives? These questions share a predictive core, but they have different targets and require different forms of evidence.

This distinction matters because relapsing multiple sclerosis has a variable course, and available therapies differ in efficacy, safety, monitoring requirements, and burden (1). Guidelines strongly endorse MRI monitoring, and new or enlarging T2Ls are widely used as evidence of breakthrough activity and treatment response, either alone or within multivariable models (2–5). The ability of MRI to detect new inflammatory activity is well-established. Greater caution is needed when the same finding is used to make a precise individual prognosis, to predict comparative individual treatment benefit, or to justify an MRI-guided escalation rule. The models and measures cited in support of these broader claims do not always provide the evidence needed for the specific task (6). Our aim is therefore not to question MRI monitoring, but to clarify what can and cannot be concluded from new T2Ls when treatment decisions are made.

Individual-level surrogacy is related to prognosis, but it is not the same as treatment-effect prediction. In randomized trials, it asks how much an earlier measure, such as a T2L count, reduces uncertainty about a later clinical outcome after the treatment effect has been accounted for. It is prognostic in aim and causal in construction. However, it does not estimate how the benefit of one treatment rather than another varies between individuals.

Shared decision-making (SDM) is different again. It is the process in which the clinician and the person with multiple sclerosis consider expected benefits, harms, treatment burdens, and personal priorities together. SDM uses evidence from detection, prognosis, treatment-effect prediction, and strategy evaluation; it does not generate that evidence.

## Discussion

### From MRI findings to treatment decisions

#### Why the four questions matter in practice

The four questions (Table 1) are conceptually distinct, but they must ultimately be brought together when treatment is reconsidered. A biomarker may be strongly prognostic without indicating which alternative treatment is preferable. Conversely, a marker may modify the relative effect of two treatments without identifying the PwRMS at highest absolute risk for disease activity or disability progression.

**Table 1:** Distinct clinical questions raised by new or enlarging T2-weighted lesions.

Clinical question	Task	What the MRI finding can contribute	Evidence required
Has disease activity occurred?	Detection	New lesions provide direct evidence of new focal inflammatory activity, provided that scans are comparable and the timing is clinically interpretable.	Reliable longitudinal MRI acquisition, lesion identification, and clinical interpretation.
What is the future clinical risk?	Prognosis	T2 lesions may contribute information about later relapse or disability, especially when combined with other relevant variables.	Externally validated models reporting absolute risks, calibration, discrimination, and uncertainty for a defined time horizon.
Which treatment is expected to be better for this individual?	Treatment-effect prediction	MRI activity may identify higher-risk individuals, but a prognostic factor does not necessarily identify greater benefit from one treatment rather than another.	Comparative causal analyses or randomized trials that assess treatment-effect heterogeneity under realistic treatment alternatives.
Does an MRI-guided strategy improve outcomes?	Treatment-strategy evaluation	An escalation rule may be useful even when prognostic discrimination is only moderate, but the consequences of applying the rule must be evaluated.	Randomized strategy studies, target-trial emulation, or decision analyses comparing MRI-guided care with relevant alternatives.

A treatment-adjustment decision should therefore consider the current evidence of inflammatory activity, the person's expected risk under continued treatment, and the expected benefits and harms of realistic alternatives. Whether a rule based on these elements improves outcomes at the population level remains a separate question. It concerns the performance of the decision strategy, not only the performance of the MRI marker.

#### MRI is one component of a multimodal assessment

Clinicians do not interpret MRI in isolation. They also consider relapses, neurological examination findings, the long-term progression of the disability, as measured by the Expanded Disability Status Scale (EDSS), as well as other clinical domains such as cognition.

Further, adherence, tolerability, age, disease duration, comorbidities, treatment safety, monitoring burden, reproductive plans, and the person's preferences are taken into account. Patient-reported outcomes and fluid biomarkers such as neurofilament light chain or glial fibrillary acidic protein (GFAP) may provide additional information, although their availability and validation for routine care remain uneven.

The meaning of a new T2L also depends on its clinical and imaging context. Relevant features include lesion location, gadolinium enhancement, spinal cord involvement, baseline lesion burden, and previous MRI activity. Research should therefore address two related questions for each clinical task: how well T2Ls perform on their own, and how much information they add to an already meaningful multivariable assessment.

#### How guidelines and clinical practice use MRI

New or enlarging T2Ls are reliable markers of active focal inflammation. Accordingly, guidelines and consensus recommendations place MRI at the center of routine monitoring, treat-to-target approaches, and no-evidence-of-disease-activity frameworks (2–5). Clinical practice reflects this emphasis. In a consensus survey of US neurologists, 81% regarded asymptomatic MRI activity as the biological equivalent of a relapse, 97% reported follow-up MRI within 12 months, and 66% would consider changing treatment after two or more new T2Ls (2).

Guidelines sometimes go further and describe MRI as a tool for predicting treatment response (4). This step requires additional evidence. Detection of inflammatory activity is not equivalent to predicting future clinical outcomes, identifying who will benefit more from escalation, or demonstrating that an MRI-guided escalation strategy improves outcomes.

#### What individual-level data show

Our analysis of individual-level data from ten clinical trials found little robust individual-level surrogacy between T2 lesion measures and later EDSS scores or relapse counts in most settings examined (7). This result should be interpreted narrowly. It challenges the strong claims that independently of treatment T2L measures precisely forecast an individual's later clinical outcome. It does not question the established role of MRI in detecting inflammatory activity. Nor does it evaluate whether acting on MRI findings improves outcomes.

A separate multivariable analysis of fingolimod response in relapsing multiple sclerosis showed moderate discrimination for relapse and new T2Ls, poorer discrimination for disability, and no detectable heterogeneity of treatment response (8). Thus, the model did not establish that the relative benefit of fingolimod could be predicted for individual PwRMS.

These findings do not imply that MRI lacks clinical value. A marker can be useful for detecting breakthrough activity even when it does not sharply distinguish PwRMS who will later relapse from those who will not. Rather, the results show that the current evidence is stronger for detection than for precise individual prognosis or treatment-effect prediction.

#### Why association measures are insufficient for individual decisions

A lesion count rarely provides a calibrated, individual probability of relapse or disability progression. Yet the measures most often reported in the multiple sclerosis literature describe association and are frequently interpreted as if they directly answered prognostic or treatment questions. This problem remains when T2Ls are included in multivariable models rather than considered alone. In four multiple sclerosis recommendation articles, association-based measures dominated the evidence used to support predictive claims (6).

Odds ratios and hazard ratios, for example, can show that PwRMS with MRI activity have, on average, a higher risk of an adverse outcome than people without such activity. They do not by themselves provide the absolute risk for a particular person. That risk also depends on the baseline event rate, the prediction horizon, treatment, and the person's other characteristics. The development of the Rio Score illustrates this distinction (9). A strong association between T2 lesions and disability progression was reported (odds ratio 8.3; 95% confidence interval 3.1 to 21.9), but the positive predictive value was only 37%. Most people classified as high risk therefore did not progress.

Predictive values have limitations of their own. They depend on how common the outcome is in the population and on how the outcome is defined. The same marker may therefore have different predictive values in different cohorts. Sensitivity and specificity also summarize performance within a defined setting, but they are not universal measures of individual prediction. External validation is needed to determine whether performance is maintained in a new population. Reported estimates should also include confidence intervals so that their statistical uncertainty is visible (6).

Confidence intervals and statistical significance must be interpreted carefully. A confidence interval around an estimated risk describes uncertainty in the estimated conditional risk, but it is not a range of possible outcomes for the individual person. A small *p*-value indicates that an association is unlikely to be explained by sampling variation alone. It does not show that the prediction is sufficiently accurate or clinically useful for an individual decision.

Categorizing lesion counts creates another problem. A threshold such as < 2 vs. at ≥2 new lesions is easy to apply, but it discards information and treats PwRMS close to the cut-off as fundamentally different while treating potentially dissimilar people within the same category as equivalent.

Finally, systematic reviews have identified many prognostic models in multiple sclerosis but few that are ready for routine use, largely because of high risk of bias, incomplete reporting, and limited external validation (10, 11). The literature contains abundant evidence of association, but much less evidence that provides calibrated and clinically useful individual predictions (6).

#### What better evidence should look like

Future studies should begin by stating the clinical question, target population, treatment context, outcome, and prediction horizon. They should report absolute risks and evaluate models in data that are genuinely independent of model development. Internal validation is necessary to assess overfitting, but external validation is essential before a model can be considered for routine care (10).

For prognosis, no single performance measure is sufficient. Calibration assesses whether predicted probabilities agree with observed outcome frequencies. Discrimination assesses whether people with higher predicted risks experience more events than people with lower predicted risks. Proper scoring rules, such as the Brier score, summarize overall predictive error. All estimates should be accompanied by appropriate measures of uncertainty.

For individual-level surrogacy, measures such as *R*-squared and the likelihood reduction factor quantify how much an earlier marker reduces uncertainty about a later clinical outcome. These measures address the precision of prognosis based on the surrogate. However, they do not establish that the marker predicts comparative treatment benefit.

Treatment-effect prediction requires a causal comparison of outcomes under alternative treatments. Methods such as inverse probability weighting, the g-formula, or g-estimation can be used to estimate treatment effects and their variation across individuals (12). The resulting models still require careful assessment of calibration, discrimination, uncertainty, and external validity, but their target is different from prognosis under a single treatment strategy.

Ultimately, treatment-strategy evaluation asks whether applying an MRI-guided rule improves outcomes compared with relevant alternatives, such as escalating treatment for everyone, for no one, or according to an existing clinical rule. This can be studied in randomized strategy trials, through target-trial emulation, or by decision analysis. Even externally validated response models may leave this final step unanswered (13, 14). Decision curve analysis can help quantify the net benefit of using a model across clinically relevant decision thresholds (15). The resulting evidence can inform, but not replace, SDM.

### Clinical implications

Three practical implications follow. First, new or enlarging T2Ls should continue to be regarded as clinically relevant evidence of breakthrough inflammatory activity. Second, clinicians should avoid translating lesion counts into precise forecasts of relapse or disability progression unless an appropriate and externally validated model supports that interpretation. This caution applies whether T2Ls are considered alone or as part of a multivariable model. Third, treatment adjustment or escalation should be based on the full clinical and paraclinical picture and discussed with the PwRMS, including the uncertainty of predicted outcomes and the expected benefits, harms, and burdens of the available treatment options.

The priority is not to reduce the use of MRI, but to strengthen the evidence that links MRI findings to individual treatment decisions. This requires multimodal models that are externally validated, quantify the incremental contribution of T2Ls, evaluate treatment-effect heterogeneity, and test whether MRI-guided strategies improve outcomes that matter to PwRMS.

## Conclusion

New T2Ls are established markers of current inflammatory activity and may add prognostic information within a broader clinical assessment. On their own, however, they do not provide a precise individual forecast, identify who will benefit most from treatment escalation, or demonstrate that a particular MRI-guided strategy improves clinical outcomes. Clear separation of detection, prognosis, treatment-effect prediction, and treatment-strategy evaluation makes the role of MRI more clinically useful and more transparent about the limits of current evidence.
